# Exploring perceptions of genetic risk and the transmission of substance use disorders

**DOI:** 10.1186/s13722-024-00470-w

**Published:** 2024-08-02

**Authors:** Amanda Keller, Emily A. Bosk, Alicia Mendez, Brett Greenfield, Carolynn Flynn, Gina Everett (DelJones), Fabrys Julien, MacKenzie Michael

**Affiliations:** 1 McGill University School of Social Work, 550 Sherbrooke Ouest Suite 100, Tour Est Montreal, Montreal, H3A 1B9 QC Canada; 2 Rutgers University, 390 George St., Room 713, New Brunswick, NJ USA; 3 School of Social Work, Boston University, 264 Bay State Rd, 02215 Boston, MA USA; 4 School of Social Work, Rutgers University, 390 George St, New Brunswick, NJ 08901 USA; 5 The Center for Great Expectations, Somerset, NJ 08873 USA; 6 Research Chair in Child Well-Being, McGill University School of Social Work, 550 Sherbrooke Ouest Suite 100, Tour Est Montreal, Montreal, H3A 1B9 QC Canada

**Keywords:** Substance Use Disorder, Genetic risk, Working Model of the child interview, New Genetics; Lay Genetics, Continuity of Substance Use Disorder; intergenerational transmission of Substance Use Disorder, Children of substance users, Substance Use Disorder Treatment, Brain Disease Model of Addiction

## Abstract

**Background:**

Substance use disorders (SUDs) have been consistently shown to exhibit moderate intergenerational continuity (1–3). While much research has examined genetic and social influences on addiction, less attention has been paid to clients’ and lay persons’ perceptions of genetic influences on the heritability of SUD (4) and implications for treatment.

**Methods:**

For this qualitative study, twenty-six structured Working Model of the Child Interviews (WMCI) were conducted with mothers receiving inpatient SUD treatment. These interviews were thematically analyzed for themes related to maternal perceptions around intergenerational transmission of substance use behaviours.

**Results:**

Findings show that over half of the mothers in this sample were preoccupied with their children’s risk factors for addictions. Among this group, 29% spontaneously expressed concerns about their children’s genetic risk for addiction, 54% shared worries about their children’s propensity for addiction without mentioning the word gene or genetic. Additionally, 37% had challenges in even discussing their children’s future when prompted. These concerns mapped onto internal working models of attachment in unexpected ways, with parents who were coded with balanced working models being more likely to discuss intergenerational risk factors and parents with disengaged working models displaying difficulties in discussing their child’s future.

**Conclusion:**

This research suggests that the dominant discourse around the brain-disease model of addictions, in its effort to reduce stigma and self-blame, may have unintended downstream consequences for parents’ mental models about their children’s risks for future addiction. Parents receiving SUD treatment, and the staff who deliver it, may benefit from psychoeducation about the intergenerational transmission of SUD as part of treatment.

## Background

### Intergenerational continuity in substance use and parental beliefs

The intergenerational continuity of substance use is well established [5–7] with multiple prospective longitudinal studies demonstrating the continuity of SUD across three generations [8]. Research on the intergenerational transmission of SUD focuses on teasing out the mechanisms and associations that underlie intergenerational continuity [4, 9, 10]. While the etiology of SUD is multi-determined, an example of developmental equifinality, heritability estimates suggest genetic factors account for approximately 50% of the risk for SUDs [11]. There has been increasing emphasis on the genetic and/or neurobiological basis of addictive behaviors in research [12], the popular press, and in many interventions as the primary explanatory model underlying SUD. A recent meta-analysis of genome-wide association (GWAS) studies involving over one million participants, has underscored the complex, probabilistic and polygenic nature of risk across SUDs and for substance specific pathways [13]. More limited, however, has been research considering how parents with SUDs understand and respond to the risk of the intergenerational transmission of SUD [11], particularly in the context of epidemiological and clinical-intervention models that emphasize genetic vulnerabilities.

Parents’ sense-making around the information they receive about addiction and its intergenerational transmission is as important as it is poorly understood. If parents come to view their children’s risk as linear, deterministic, and inevitable (e.g., ‘in their genes’), a self-fulfilling prophecy may be set in motion with the power to shape their investment in and motivation for treatment, parenting behavior and, in a transactional manner, parent-child relationship patterns across a child’s development [14, 15, 16]. Linear and deterministic beliefs may then impact parental mental models of their child’s future and contribute to negative perceptions and downstream effects on parenting behaviour. Conversely, parents who understand their children’s risk for later addiction to be probabilistic, and modifiable may actively seek intervention, be more engaged in treatment, or attend to environmental and parenting factors that may be more amenable to treatment and also influence substance use, in order to prevent future addiction for their children.

Whichever the case, we have relatively little empirical knowledge about how parents with SUD conceptualize the impact of their ‘personal histories of inheritance’ on their young children’s future risk of developing a SUD. Although many studies examine the etiology of continuity of SUD within families [4, 8–10, 17], there is less research that examines how parents with a SUD understand and respond to the risk of intergenerational transmission [14, 18, 19]. This work seeks to fill this gap, building a theory about parents’ understanding of their children’s risk for the later development of a SUD. Examining the sense-making [17] of parents in SUD treatment about the risk of intergenerational transmission of SUD helps identify how families interpret and then respond to risk for conditions that have complex etiological origins.

### Explanations for the intergenerational transmission of substance use

Explanations for the intergenerational transmission of SUD are multi-factorial. Decades of research point to contributions of genetics [9, 12], family interaction patterns [20–23] and family and community norms [24–26] as influencing the continuity of SUD in families. These different explanatory factors are examined in more detail below.

Briefly, twin studies demonstrate genetic links related to the quantity and use of specific substances. More recent research considers the genetic risk of SUD to be linked to sensitivities in the Brain Reward Cascade, which controls the release of dopamine [24]. Deficiencies or variations in this system of neurochemicals may heighten the individual risk of SUD. While some studies have not shown an association between specific genes or neurochemical clusters, others assert that anywhere between 25 and 75% of risk for the development of SUD is attributable to these genetic causes [4, 24]. Research in genetics and neurobiology suggests strong associations between neurochemical vulnerabilities and later development of SUD. These associations, however, are probabilistic with great variation in the strength of the associations and genetic researchers employing GWAS strategies to identify loci probabilistically important to SUDs, have highlighted the challenge and limitations of the polygenic nature of substance use disorders [27]. For example, the Virginia Twin study demonstrated that while genetic vulnerabilities were a factor in the development of adolescent SUD, familial and peer-related factors were more likely to influence later use than genetic vulnerabilities [25]. Similarly, a Swedish study examining adopted and non-adopted children of parents with SUD found that adopted children who received sensitive and consistent care were less likely to develop a SUD than children raised by their biological parents [26]. These studies make a strong case for how environmental influences transact with genetic vulnerabilities to shape later development of a SUD. Genetic predispositions to SUD are, therefore, probably best understood in dynamic relation to their environmental and other contextual factors.

Children’s exposure to parental substance use can occur in multiple forms in addition to genetics: children can be exposed chemically to substance in utero and exposed socially through parental use at any stage in the life course [28, 29]. The impact of prenatal exposure to substances varies depending on the substance, dose, frequency, and timing of use during pregnancy [30]. However, research suggests that social conditions, particularly socio-economic status, parental mental health, and parenting style, are more likely to influence child outcomes than chemical exposure to substances itself [31–33]. Therefore, the impact of prenatal chemical exposure on negative child outcomes cannot be attributed solely to neurobiology and varies widely.

Adult SUD often co-occurs with trauma, mental health challenges, family stress, and poverty [34] which can also underlie the development of a SUD. Trauma, mental health, poverty, and addiction are often chronic, cumulative, and reciprocally reinforcing. When trauma, mental health challenges, poverty and parental SUD co-occur, risks for negative child outcomes, such as the later development of a SUD, increase [35]. Teasing out the relationship between genetics and environmental influences is particularly complicated because parental trauma, mental health challenges and SUD independently, and interactively, can create challenges in the caregiving environment that shape children’s development, which, in turn, can influence their later development of SUD.

There is a robust body of research that considers parenting behaviors among caregivers with a SUD. Parents with a SUD who have young children are more likely to struggle to provide sensitive and consistent care, have inappropriate developmental expectations of their child(ren), have inconsistent rules or limits, be reactive, and engage in harsh parenting [36–42]. Each of these parenting behaviors are associated with poorer quality parent-child relationships and poorer child outcomes [43–47]. Parents with older children who have SUD are less likely to monitor their older children, are more likely to struggle with limit setting, and are more likely to have lower levels of positive interactions [48]. These parenting behaviors create relational vulnerabilities that make the future development of a SUD more likely for a child.

The association between the caregiving environment and the later development of a SUD is born out in the extensive literature linking parental SUD to negative child outcomes. Having a parent with a SUD is associated with poorer outcomes for children and youth across the life course [5, 49]. The risk of intergenerational transmission of SUD, and of adverse childhood experiences and later outcomes increase when both parents have a SUD [50–52]. Young children whose parents have a SUD are more likely to have behavioral difficulties, poorer physical health, and developmental delays [53]. Youth who have a caregiver with a SUD are more likely to have symptoms of internalizing [54] or externalizing mental health disorders [54, 55, 56]. Viewed from a transactional perspective, these poorer outcomes become risks for the later development of a SUD as youth may be more likely to engage in risky behaviors or seek relief from negative feeling states that result from early childhood trauma and/or family dysfunction [28, 57–60]. Challenging parenting behaviors and impingements in the caregiving environment may create relational vulnerabilities that make the development of a SUD more likely. Therefore, the emotional legacy of challenging parent-child relationships is another mechanism by which the intergenerational continuity of SUD takes place.

### Primacy of brain disease model of addiction (BMDA) in treatment and support programs

Even as studies focus on the interplay between neurobiological, familial, social, and structural factors that underlie the development of a SUD, there has been an increasing focus on the Brain Disease Model of Addiction [61–64] as the primary explanatory model for addiction in public-facing treatment and support program offerings. This etiological explanation was reinforced by its association with researchers at the National Institute on Drug Abuse and by the American Society for Addiction Medicine, which set research and funding priorities related to examining addiction as a chronic brain disease [4, 36, 63].

The BDMA holds that addiction is an unwanted condition where initial, voluntary substance use gradually becomes involuntary as the brain’s reward system becomes ‘hijacked’ [65] via a set of neurochemical processes. In this ‘hijacking,’ a negative feedback loop occurs where increased exposure to substances and their associated positive feelings (reward) becomes dulled after repeated exposure. Conditioned to receive a positive response, people seek higher doses of a substance and use substances more frequently when the expected reward is not received. Increasing the quantity and frequency use of substances also produces changes in the stress response system through alterations to the amygdala. The stress-response system becomes highly reactive, which in turn, can make it harder to manage day to day challenges. Not only does the process of addiction decrease the release of dopamine in relation to the use of the targeted substance, over time the brain becomes less sensitive to any experience that releases dopamine) and the pleasures associated with it [66]. The impaired reward and stress systems interact, producing ever-increasing triggers for use as people become conditioned to seek an increasingly difficult to obtain reward [66]. This occurs while the pleasures of everyday life become more attenuated and stress and negative experiences increase, creating a greater need to use substances [66].

Changes in the neural reward and stress response circuitry combine with changes in the neural regulatory circuitry to create the basis for addiction. According to the BDMA, addiction overwhelms areas of the brain related to executive control which governs decision-making, inhibitory control and self-regulation. Disruptions in the prefrontal cortex make it difficult for an individual to exercise choice in their use and achieve or maintain sobriety [57, 60].

The social, practical, and scientific consequences of the BDMA as an explanatory model have been widely debated [4, 30, 58]. Proponents contend that a neurobiological explanation of addiction will decrease stigma, create treatment options through the development of medications to block cravings, and offer a clear scientific explanation for why people continue to use drugs when the effects are no longer pleasurable and the consequences of use severe [63, 66]. Critics argue that the BDMA model may lead people to believe that addiction is intractable, and the unintended consequence of this framing is that it will decrease interest in treatment and impede self-efficacy [67, 68]. Further, the static presentation of the brain in the BMDA model, critics assert, is contradicted by more recent understandings of neuroplasticity and the adaptability of the brain [36]. Critics also contend that there is very little empirical evidence to support the BMDA as an explanatory model, noting that in practice, BMDA has not led to significant policy or treatment interventions that alleviate addiction [69].

Despite the substantive disagreements about the empirical basis and utility of the BMDA there is little disagreement over whether this model of addiction holds primacy both scientifically and in the popular imagination. Research examining the impact of the BMDA on doctors, addiction treatment providers, and the public suggests that neither the most positive or negative impacts predicted by proponents or opponents have come to pass [4, 64, 68]. The explanatory dominance of the BDMA makes it important to examine its implications for personal decision-making and sense-making related to the intergenerational continuity of SUD. As demonstrated above there are multiple contributions to the intergenerational transmission of use. How people understand these contributions is likely to matter for how they approach the intersection of SUD and parenting.

### Lay understandings of genetics and stigma

Social science examinations of lay people’s meaning-making of genetics and disease makes clear that interpretations of the scientific literature are not monolithic and often do not show concordance with expert theories [4, 67]. Rather, how people make sense of disease, genetics, and risk varies by person and the social acceptability of the disease itself [70–75]. People demonstrate a profound capacity to hold complex and contradictory views as they interpret and then apply scientific literature to their own lives and decision-making. Often these applications belie the more concrete presentations in the research as people adapt neuroscientific or biological understandings of disease to fit within their other understandings of themselves in dynamic and nonlinear ways [68, 70, 76].

Personal narratives about genetic inheritance and addiction complicate academic debates between BDMA proponents and opponents as predictions about the promises and the pitfalls of the BDMA are not always borne out. Research on public understanding of the BDMA model suggests varying degree of acceptance and reduction of stigma. In one study, while half of the participants accepted a brain disease explanation of addiction, only a small fraction then linked this etiology to a reduction in moral judgement [68]. Dingel and colleagues found that among participants in an addiction treatment program, most did not find the genetic conception of addiction etiology helpful, but a minority of participants reported that it released them from feelings of shame and guilt related to their substance use [4]. Dingel’s research demonstrates that there is not a singular response to genetic or biological narratives about addiction.

Attribution theory describes one rationale for asserting that a neurobiological conception of addiction will reduce stigma. Attribution theory suggests that “low causal responsibility for a stigmatized characteristic […] is associated with less blame and more positive emotions” [77]. In other words, the blame for an outcome (for example, having a SUD) will decrease if the behavior is attributed to a cause over which a person has little to no control [75]. However, when the behavior is seen as untreatable and the affected person is seen as likely to cause harm, stigma may increase [78]. Schnittker found that people combined explanations of SUD so that genetic and neurobiological explanations of addiction rested alongside one another [76]. This research provides support to the idea that the process of medicalization for stigmatizing phenomena does not always provide a simple route for decreasing negative consequences of these conditions [79]. Another unintended consequence of genetization is that individuals who believe their condition has a genetic cause may not believe they have any control over modifying its trajectory or their own outcomes [70]. Discussing genetics odds ratios with families at risk of any condition is a complex endeavor because there is always the risk of misinterpretation, which could come from a variety of factors such as psychological issues in the families themselves [80], media portrayal of common conditions, as well as a failure to grasp the nuances of epigenetic moderation and environmental contributions. Finally, there is a distinct possibility that these discussions conducted without appropriate caution and care could stigmatize already vulnerable individuals and families [77].

### Parental representations and genetic anxiety

No studies, to our knowledge, consider the relationship between parental representations of children, internal working models of attachment relationships, and their sense-making of genetics. To understand how these concepts may be practically linked, it is first necessary to review the fundamentals of attachment theory. Beginning with John Bowlby, attachment theorists have recognized that there is an explicit connection between a parent’s mental representation of relationships based on their past relationships and their representations, perceptions, attunement, and interactions with their own child. Bowlby articulated that these mental representations serve as *internal working models*, which have important generalizable and predictive power [81–85]. Fundamentally, internal working models are dynamic representations developed in the context of an interactive experience between a primary caregiver and an infant when an infant is distressed [86].

Attachment styles emerge from the internal working models that develop from these patterned interactions. The four attachment styles are secure, avoidant, ambivalent, or disorganized [44]. Secure attachment styles develop from distress being sensitively and consistently responded to. Anxious attachment styles refer to the caregiver responses to infants’ distress in ways that amplify it or distress the caregiver. Avoidant attachment emerges from ignoring or dismissing a child’s distress. Disorganized attachment occurs when a caregiver’s responses to a child’s distress are unpredictable and inconsistent. Attachment patterns matter because they become enacted as relational and emotional regulatory strategies throughout life, serving as a template from which people engage with and interpret the world.

The Working Model of the Child Interview (WMCI) is a widely used structured clinical interview that identifies parental representations, or internal working models, of their children and their relationship to them. Coding for the WMCI results in three reliably distinct typologies: Balanced, Disengaged and Distorted [87]. Narratives of *Balanced* parents exhibit an investment in the relationship with the child with an ability to appreciate the child’s subjective experiences and openness to the child’s individuality. In contrast, the *Distorted* typology evinces narratives often marked by inconsistent and at times incoherence and signs of an inability to focus on the experiences and characteristics of the child as separate from the adult’s own preoccupation with other concerns. Narratives from *Disengaged* parents are often marked by an emotional coolness, distancing, or indifference to the child. These working model typologies map onto infant clinical status [88], and attachment classifications with balanced maternal representations linked to secure infant attachment, disengaged representations linked to avoidant attachment and distorted representations linked to anxious attachment typologies [89].

Parental representations of their child, schemas about the world, and emotional regulatory strategies may influence how they make sense of genetics and their child’s potential inheritance of less desirable conditions, like SUD. It may be that as parents with avoidant or disengaged representations are more likely to be deterministic or concrete in their conceptions of the future or be so overwhelmed by worry that they avoid or dismiss these anxieties, consistent with an emotional regulatory strategy to generally avoid distress. It may also be that parents who have distorted representations of their children also have distorted representations about the likelihood that their child will develop a SUD in the future. For parents with balanced representations of their children, it may be that interpretations of genetics and the intergenerational transmission of SUD may be similarly balanced between concern and openness to the unknowability of the future. The representational aspects of lay peoples understanding of SUD, particularly as they relate to continuity of SUD across generations has yet to be examined.

## Methods

### Participant recruitment

This research project is a study nested within a larger project examining the implementation and efficacy of a family-focused SUD intervention into a residential treatment center that houses mothers and their children ages 0–5. This data comes from 26 culturally and ethnically diverse mothers aged 18–42 who were living in a residential treatment facility for mothers with SUD and their children under age five. The treatment center is in the Northeastern United States. For this study, twenty-nine in-depth, structured Working Model of the Child Interviews (WMCI) were conducted with mothers receiving inpatient SUD treatment.

### Working model of the child interview

The WMCI is a structured 19-question interview used to assess parents’ representations of their attachment and relationships to a particular child [89]. The WMCI is designed to gain insights into how parent perceives and understand their relationships with their children. The 19 questions aim to elicit information about the child’s thoughts, feelings, and beliefs regarding their attachment figures. These questions are carefully crafted to assess various aspects of the child’s attachment-related representations and cognitive processes.

### Data collection

The WMCI interviews took place in the treatment center and lasted approximately 60 min per participant. Three interviews were excluded due to incomplete interviews (e.g., if all 19 questions were not asked). Most interviews were completed on the same day. Three interviews were completed over two days due to client fatigue or caregiving responsibilities. This left a total of 26 interviews for analysis. Interviews were audiotaped and transcribed verbatim for analysis.

### Data analysis

The 26 interviews were first clinically coded by two trained WMCI coders. Participants were identified as either Balanced, Disengaged or Distorted in their parental representations of their child. A complete review of the widely-used WMCI coding procedures and further description of the classification typologies is beyond the scope of this paper, but have been described at length elsewhere [90]). During clinical coding, an unexpected theme surfaced, with many mothers spontaneously stating, without specific prompt, that their primary concern was their children’s purported genetic risk for addiction. These concerns were raised by parents despite no reference to genetics or even addiction being included in the interview questions or prompts. Once this theme emerged, we entered the interviews into Qualtrics to code and count the number of participants who mentioned genes or genetics during their interview. Once we understood close to a third of participants expressed concerns specifically about genes or genetics during their interview, these interviews were then qualitatively thematic analyzed to examine maternal about the intergenerational risk of the development of SUDs.

To analyze the 26 interviews, we used NVivo 10 software to aid and organize the qualitative data analysis. We utilized a modified grounded theory approach. Initially, we conducted an inductive coding process to identify emergent themes in the data based on our initial observation of parents concerns about their child’s risk for future addiction. Through this process, we developed a set of codes that captured the different concerns expressed by the participants regarding the intergenerational continuity of substance use and its potential impact on their children. Some examples of these codes are genetic anxiety, the child may develop a substance use disorder, unwilling to think about the future. Once these codes were established, we deductively coded the interviews to examine the presence of these codes. This process was carried out collaboratively between the co-authors over several weeks. After the interviews were deductively coded, we established the theme of genetic anxiety, which we defined as mothers expressing anxiety about their child inheriting a SUD. Examples of codes we used throughout the analysis from which we identified larger themes were genetic anxiety (when genes or genetic was specifically mentioned), difficulties thinking about the future, and fear of intergenerational substance use (without mentioning genes). We utilized several strategies to establish rigor in our analysis [91]. Specifically, once the codebook was established by the first, second, fourth and fifth authors, disagreements among coders (first, fourth, and fifth authors) were resolved using a consensus coding process. Multiple coders coded the same transcripts to ensure inter-rater reliability and the objective presence of themes. Additionally, coding was reviewed by the second author to establish inter-rater reliability further. The first and second authors also used negative case analysis to test emerging theories and as a check against confirmation bias.

## Results

### Demographics

The present study examined a sample of twenty-six mothers who ranged in age between 18 and 42. The sample displayed a relatively diverse racial and ethnic composition. Among the participants, 61% identified as white, 12% identified as Black, 8% identified as multiracial, and 1% identified as Asian. One participant chose not to self-identify their racial background, and the rest identified as other. The large coupling of “other identified persons” is likely accounted for by the 27% of the participants who identified as having a Latina/o ethnicity. In terms of marital status, 20 participants reported being single, while 6 participants were married or had a significant other. Regarding employment status, 21 participants were unemployed at the time of the study, while 5 participants reported being employed. Concerning participant’s educational background, 1 participant had some high school education, 4 participants had a high school diploma, 9 participants had some college education, and 12 participants’ educational background was unknown. They all spoke English as their primary language.

### Anxiety about their children’s future

Our analysis of the interview data revealed that when asked about their children’s futures many parents with SUDs had anxiety about their children’s risk to develop SUDs. These themes emerged through answers to the following two standardized questions: “What do you imagine your child will be like as an adult?” or “What is your biggest concern for your child’s future?” When asked this general prompt, 54% of mothers spontaneously expressed specific concerns that their children might go on to develop a substance use disorder (SUD). Additionally, 37% of mothers expressed reluctance or difficulty imagining their children’s futures. Perhaps most strikingly, 29% of mothers spontaneously reported unprompted specific concerns about their child’s genetic predisposition towards addiction. Many of these concerns related to their children’s genes show important misunderstandings about genetic risk factors critical for substance use professionals to understand and consider in their clinical work.

### General substance use anxieties

Most mothers (54%) in this study reported some anxieties about their young children’s propensity to develop addictions and maladaptive behaviors, as these four different participant parents elaborated:

Probably just being a troublemaker and not be into things that could get him in jail with the bad crowd and drugs.

Don’t know what he remembers from being a little baby, but there’s definitely… Besides just being around it, I’m concerned that he has some of the isms of it all, if that makes sense. Like just some of the behaviors with the emotional and erratic behavior that he has. I know that I was like that.

She’s still too young for me to find that out but that’s something that you have to worry about. If I see she gets older and I see that everything I give her that she gets addicted to or abuses, and that’s something to worry about.

I am worried that he might grow up to suffer with alcoholism.

These concerns indicate the pervasive worry among mothers about their children’s vulnerability to addictive behaviors and negative influences, and in some mothers reflecting a desire to steer them away from potential pitfalls and towards healthier paths of development.

### Genetic anxiety

The concerns about genetic anxiety varied from more deterministic to more uncertain to more realistic understandings about the genetic influence on addiction and the risks for the intergenerational transmission of addiction. The quotes from three mothers below exemplify range of more deterministic worries about the genetic influence on addiction:

“I fear he will be tempted and tested with drugs and alcohol. He probably already has the gene, but I fear that it might take him.“

“My fear is that she could have the genetic trait of addiction.“

I have so much fear about her future because of her genetics and because of her experiences in life that it’s terrifying.

These statements frame addiction as originating within a single gene or set of genes, lying dormant until one day when they spontaneously become expressed. In contrast, still other participants framed this risk within the complex array of social, community, or familial influences. For instance:

My fear for her is that she might get in with the wrong crowd, or be promiscuous, or experiment with drugs or become addicted to drugs. In this day and age, there’s just so much. And to be predisposed to it scares me.

Some of it is, again, stuff that I don’t know, like even when I was pregnant worrying genetically his predisposition to have these issues, but he’s grown up, his immediate caretakers, besides my parents, are addicts and alcoholics. Whether active or not, there’s still something, like a component to type of person that that is, you know what I mean? What am I playing with here? So, I don’t know.

I worry for her teens because I know what her dad and I have I know our history, so I know what she’s coming up against with the stigma plus genetically. And I see what we’re going through with our 15-year-old, so I worry for her when she becomes a teenager.

These unprompted discussions of genetic risk capture a significant anxiety which was unexpected in these mothers and demonstrate the range of attributions mothers made about the transmission of SUD.

### Addiction signs in toddlers

A few mothers reported that they believed their children, all under age five, already had addiction traits or displayed certainty that they were destined to inherit their SUD. For example, three mothers expressed the following worries:

Most of all, I fear that she’s going to be an addict. Because I already see addict behaviors in her. Already. Just with my family history, she’s got a solid 75% chance that she’s going to struggle with substance abuse.

I was worried because both the parents, both his father and I are addicts and alcoholics, so definitely I was like, ‘All right,’ it’s like the baby isn’t even born yet and I’m like, ‘Oh my God, I’m going to have to send him to rehab,’ it’s really not. A little bit of thinking too far ahead there.

I’m definitely worried about the whole addiction thing. I tell people that when I was pregnant and when he was first born. They’re like, ‘Relax, he’s not even drinking. The only bottle he’s drinking out is like a baby bottle now,’ but his father is an Irish alcoholic by blood, and I have had nonstop issues throughout my life.

These last few statements reveal a more reductionist and deterministic narrative about their children’s future trajectories that the mothers assigned based, in part, on their family history. While the mechanism by which SUD is transmitted intergenerationally is likely multi-faceted and complex, the primacy of the concerns about genetic risk and children’s risk for development of SUD are important try to understand and address in treatment.

### Challenges discussing the child’s future

In contrast to mothers who worried about the genetic or intergenerational risk of SUD for their children, 37% of mothers had difficulty envisioning or discussing their child’s futures when directly asked what they imagine their child will be like as an adult. These difficulties in imagining the future ranged from more positive sort of twelve-step ‘one day at time perspectives’ to more fearful based avoidance of negative thoughts.

I don’t know. I can’t imagine him there yet. Again. I can’t even picture it yet, but I hope that I’m able to give them a good enough life that they deserve, both my kids.

I don’t know. I don’t know what she’s going to be like. Right now, she’s fun. She wants to explore. She wants to do different things, which is great. And I love that she wants to know things. So, I’m not really sure how she’s going to be. You never know.

“I ain’t thinking about that right now. No, I don’t want to rush the process.“

Well, I don’t know how she’s going to be at future ages. I don’t know. Maybe I would prefer her at three.

“I have to say … I’m sorry. My worst fear is him; I don’t know, I don’t want to think nothing bad. I don’t see anything. I don’t know, I try not to think of nothing bad. I don’t know. I can’t say.“

Honestly, to put it quite frank, if she survives into adulthood, I would be surprised. Shocked. I mean, I have hope, obviously, but….

These statements highlight some mothers’ reluctance to imagine their child’s future. While we don’t know why some women can’t or prefer not to imagine their children’s futures, it is important to highlight that most parents who did not report anxiety about their children’s futures also shared in one way or another that they did not want to think about it.

### The connection to internal working models

To explore these potential connections further, we mapped the attachment typologies from the WMCI onto the genetic and substance use anxiety coding to understand if there were any connections of potential future clinical importance in need of additional study.

### Working model findings

We analyzed the WMCI classifications and, in this sample of 26 mothers with SUD, 50% (*n* = 13) were coded as having a Balanced working model representation. In contrast, 38% (*n* = 10) were coded as having Disengaged styles, and 12% (*n* = 3) were coded as having distorted working model patterns.

**Fig. 1:**
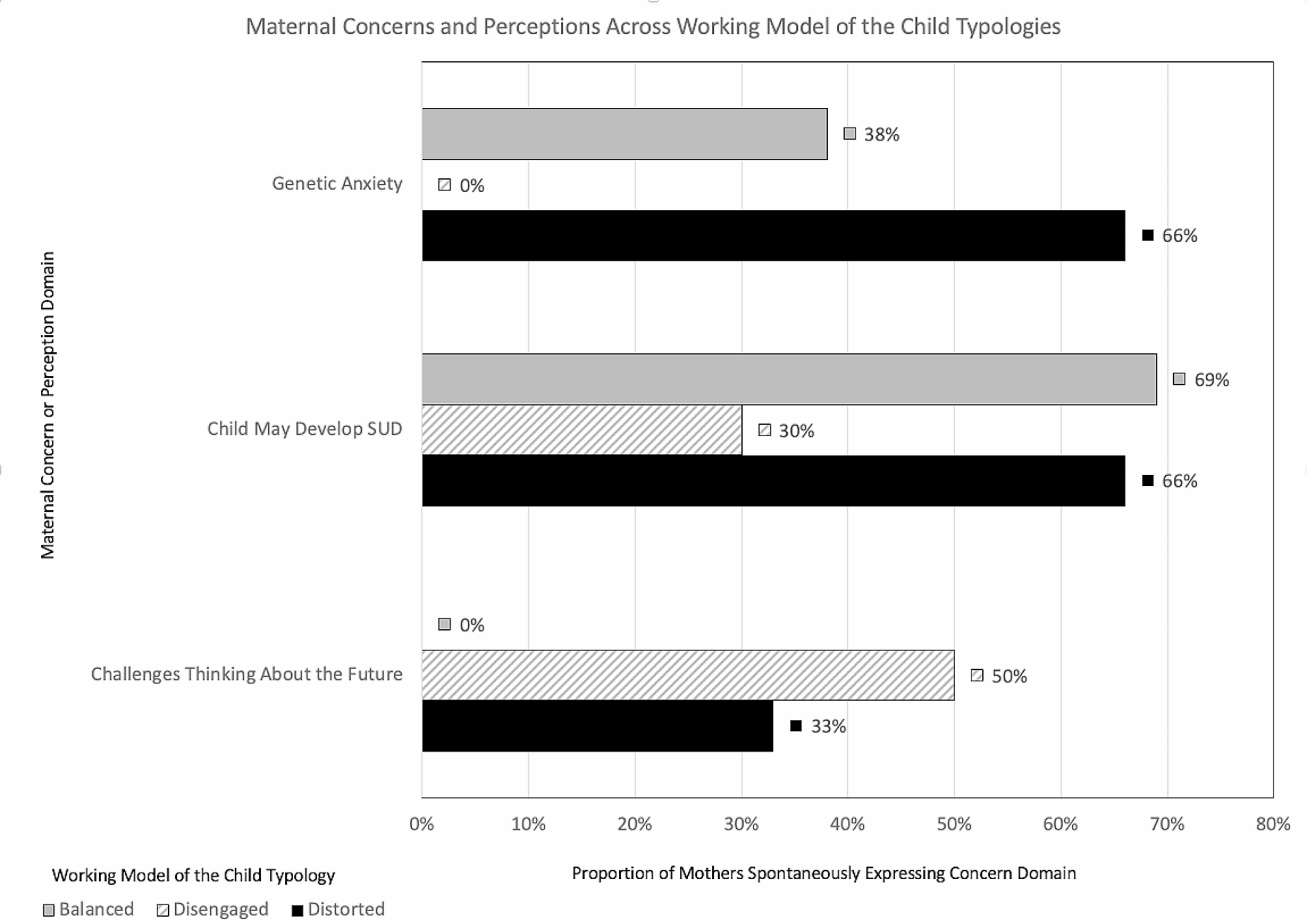
Maternal perceptions and concerns about their child’s future mapped on to the Working Model of the Child attachment classifications

We then mapped the attachment classifications in this sample onto maternal reports of worry or concern around their children’s futures (Fig. 1). The mothers coded as having balanced attachment profiles were more likely to be able to discuss their anxieties about their children’s future during the interview. At the same time, none of the mothers with disengaged attachment profiles shared concerns about their children’s genetic risk. Mothers coded as Disengaged were also less likely to report problems about their children’s future SUD risk factors than mothers showing Balanced or Distorted attachment styles. Mothers with Disengaged profiles, however, were more likely to state that they did not want to consider their child’s future or be unable to discuss their child’s future when prompted.

## Discussion

The present study delves into the nuanced perspectives of mothers undergoing substance use treatment, shedding light on their perceptions of intergenerational transmission of addiction risk. The findings underscore important anxieties for parents with SUDs with a range of presentation, with some mothers sharing their concerns about the future openly, while others experienced difficulties imagining or discussing their children’s futures. Furthermore, a few participants expressed a prevailing belief in the existence of a singular “addiction gene,” or concerns about their child’s genetic predispositions despite the absence of scientific consensus on such a genetic determinant and even more participants expressed general anxiety about their young children’s future likelihood to develop a SUD. Parents with SUD anxieties about the intergenerational transmission of addiction in their families likely represents an under addressed area for treatment and research.

Addictive behaviors run in families, with estimates suggesting that children of people with SUD are four times as likely to develop a substance use problem themselves [92, 93]. However, the development of SUD is not a straightforward genetic process [94]. Intergenerational transmission is just as likely to be influenced by relational and social experiences as genetics. Yet how mothers understand intergenerational transmission of addiction is important because at least some expressed overly simplified beliefs about their child’s genetic predispositions when they were asked open-ended questions about their thoughts on their children’s futures. Furthermore, that 54% of mothers spontaneously reported they were worried their child may develop an addiction, highlights the prominence of these concerns about intergenerational transmission of SUDs, and that they are holding beliefs about explanatory models.

The notion that addiction is genetically determined is a fascinating perception that merits careful consideration in program design and practice, with real implications for adherence and motivation for treatment for parents who may hold deterministic understandings of these polygenic and probabilistic associations. While scientific research has illuminated the complex interplay of polygenic associations [94] and environmental factors in addiction susceptibility, lay public perceptions of both staff and clients alike in treatment programs of linear causal mechanisms, or even of single genes responsible for addiction, remains an oversimplification. It is crucial to underscore the modifiability of addiction as a condition and emphasize the remarkable plasticity of the human brain. By highlighting the malleable nature of addiction and the brain’s capacity for change, we may challenge the static presentations of addiction as an inevitable outcome determined solely by one’s genetic makeup. This reframing has the potential to open avenues for exploring how interventions targeting cognitive, behavioral, and social aspects of addiction can empower individuals to exert agency over their recovery journey.

A strategic approach to engaging clients’ genetic attributions can prove instrumental in enhancing treatment completion rates and fostering meaningful progress. Encouraging people to engage in reflective meaning-making around their genetic and intergenerational beliefs and concerns can serve as a catalyst for self-efficacy and resilience. By collaboratively unpacking and addressing familial histories and deterministic ideas about addiction within the therapeutic context, clinicians can empower clientss to reframe their perceptions of genetic influence and recognize the multifaceted nature of addiction development. Integrating these multifaceted theories into substance use treatment not only aligns with contemporary scientific understanding but also offers a pragmatic framework for enhancing treatment engagement, reducing stigma, and nurturing a more comprehensive view of addiction and its potential for transformation.

We suspect these concerns are more widespread among this client population than our data can report for two reasons: First, mothers were not directly asked about their children’s risk factors for addiction or their understanding of SUD etiology. If asked directly, more mothers might have raised these concerns. Second, 37% of mothers had difficulties even discussing their children’s futures. This response is open to interpretation, as the reasons mothers wanted to avoid thinking about their children’s futures likely vary among participants. However, many participant responses indicate that this avoidance is likely an alternative manifestation of anxiety.

Working models and expressions of anxiety related to children’s future risk for a SUD appear interconnected. Mothers with balanced working models of their children were notably more inclined, or able, to express their anxieties about their children’s future during the interviews. Conversely, none of the mothers with Disengaged profiles expressed concerns about their children’s genetic risk. Moreover, mothers with Disengaged attachment styles were considerably less likely to report worries about their children’s future SUD risk factors than mothers displaying balanced or distorted working models.

In a clinical population struggling with SUD, attending to anxieties can help improve treatment outcomes, as existing research has sufficiently established that anxiety symptoms affect SUD and the reoccurrence of substance use [95–97]. Anxiety manifests in a continuum ranging from a protective level of concern to an overwhelming level that causes a freezing or avoidant response [98]. Therefore, for mothers concerned about intergenerational SUD and epigenetic risk factors, this anxiety may benefit some of them by motivating a focus on their own recovery needs while also allowing them to maintain an awareness of necessary preventive efforts to mitigate the transmission cycle and focus on opportunities for discontinuity in transmission through the work they are doing. However, mothers who assume SUD is inevitable in their children, as well as those who avoid all thoughts of SUD transmission, may be less attentive toward their children’s risk factors and may fail to intervene when warranted. Therefore, clinical practice with parents with a SUD should routinely address genetic misconceptions and parenting anxieties. Family-focused SUD programs that focus on treating both parent, children, and their relationship would particularly benefit from an explicit focus on these issues, which may weigh on parents and be outside the awareness of treatment providers.

### Clinical implications

Three key features of this data are relevant for clinical practice. First some mothers believed they or their children had “the addiction gene.” Since there is no single addiction gene, this statement represents misinformation. A small but possibly consequential percentage of these mothers believed that their infants and toddlers already showed signs of addiction, which could create a self-fulfilling prophecy. Mothers sharing concerns about how quickly their “addict behaviored” infants feed from the bottle and those imagining their toddler needing inpatient drug use rehabilitation in the future have unaddressed anxieties or projections that could impact their parenting and the parent-child relationship. Parallel studies of women experiencing domestic violence during pregnancy have demonstrated that un-balanced WMCI typologies are predictive of the experience of violence shaping perceptions of fetal behaviour, perceiving even signs of kicking after quickening as indicative of fetal aggression or propensity to later violence perpetration [99]. Finally, 37% of mothers in this sample were unable or unwilling to imagine or discuss their children’s future selves when prompted, suggesting that some have overwhelming parenting anxieties that could benefit from finding space for expression and reframing. These issues are under-examined in research and underappreciated in clinical practice.

Understanding addiction or trait transmission among mothers in active treatment for SUDs is important as sense-making around addiction and intergenerational transmission may influence important life decisions and critical relationships. For example, studies have shown that SUDs can affect marriage and childbearing decisions [14]. In one study, 5% of substance users report an unwillingness to have children due to the fear of passing on their addictions to the next generation [14]. Some qualitative research on the genetics of psychiatric illness has documented fatalistic thinking about genetic risks in the general population [100]. These beliefs are likely further intertwined with parents’ attachment styles in ways that have yet to be determined or fully investigated. When people’s sense making about addiction, relationships, and intergenerational transmission is left unexamined it is also left unaddressed. Psychodynamic examination of people’s schemas for addiction and their children are critical aspects of the intersection between parenting and substance use that would likely benefit from more explicit attention in treatment models.

The information sources from which the mothers in the present study have derived their beliefs about addiction risk factors and “addiction genes” remain unclear and are likely multi-determined. These sense-making narratives around addiction etiology may be passed from staff to client, client to client, popular media to participant [101], or some combination thereof. The narratives surrounding the intergenerational paths of substance use misalign with the more nuanced transactional and multiply determined understanding of addiction in the current research literature. Clarifying the multi-factorial and more nuanced models for the development of SUD may be an essential and missing component of SUD treatment. A clearer understanding could alleviate distress by reframing risk perceptions and refocusing attention towards opportunities for support and intervention.

Therefore, service recipients may benefit from psychoeducation efforts to alleviate their anxieties and prevent misunderstandings. Kalb and colleagues reported that their clients are interested in receiving genetic counselling about substance use disorders but that this population currently lacks adequate and specific service offerings [14]. Genetic testing or intergenerational counselling initiatives must be undertaken sensitively [18], with adequate ethical consideration. As Dingel points out, racialized and minoritized people may be sensitive to being told the issue is genetic, which they may interpret as an insult to their community of origin [4]. Moreover, addictions are also more common in low-resourced communities [102]. It would be insensitive for clinicians or lay staff from well-resourced communities to assert generalizations about the genes of lower-income people. Therefore, while we cannot know with any certainty where this message and model of addiction is coming from, our paper highlights the issue and suggests that addiction professionals use caution and that providers, both professional and para-professional, as well as the increasingly called upon peer-navigators, would also benefit from training on more nuanced messaging for clients about the probabilistic nature of risk and the environmental and social domains amenable to treatment and support. As our findings demonstrate, misunderstandings and over-generalizations can lead to unintended anxieties and potentially intergenerational consequences. Furthermore, our data suggests that how clients interpret and react to this information is likely influenced by their attachment style and familial histories with addictions. Practitioners must ensure that facts are communicated accurately, ethically, and sensitively.

### Future research directions

Future research may focus on parents’ perceptions of SUD transmission within diverse settings. How many parents with SUDs are anxious about substance use transmission, and with whom do they share these concerns? Evaluating whether these perceptions could impact parent-to-child transactions would also be essential. Does the parenting belief that a child will develop addictions have the potential to impede the development of healthy relationships with children? Does potential anxiety about passing on addictive behaviors to children contribute to shifting parenting behaviors – either in the direction of increased intrusive parenting practices to over-compensate for anxiety about the child’s predispositions or in withdrawal and distancing behaviors? Do parents who believe that their child will develop addictions tend to come from families with higher rates or more dysfunctional patterns of addictions? Future longitudinal research has the potential to elucidate the directional chaining of these transactional program-parent-child processes and thus develop potential ports of entry for intervention.

Critically, researchers must examine psychiatric, pediatric, psychological, social work and other allied health-professionals’ and lay staff’s understandings and discussions about the etiology of addiction with clients, as we believe that important information is likely being lost in translation. A careful review of how the role of genetics and environment are being presented to mothers in SUD treatment is warranted. Misunderstandings about the etiology of addiction is likely to cause increased anxiety in a subset of clients and additional research could help clarify and address how more complex integrative models of addiction could be developed to accurately and sensitively explain why addictions run in families and what actions can be taken to mitigate their intergenerational impact. Additionally, we have no understanding of how children whose parents have an SUD, make sense of their own risk for developing an SUD themselves as they transition through middle childhood and into adolescence and then adulthood. Similar misinformation, confusion, and anxiety may likely be influencing risk assessment and decision-making among youth. This is one unexplored area that could use further examination.

Given the ethical and clinical implications of propounding causal theories such as the brain-based disease model [63], clinical practice may benefit from interdisciplinary teams of genetic counselors, psychiatric professionals, psychologists, social workers, experienced peer addiction workers, and neuroethicists to explore these issues more fully at all program design, development, and implementation stages. What should we explain to clients, when, and how? The harms and benefits of explaining probabilistic risk factors using various models must be considered and debated in consideration of the clinical needs, potential harms, probable misunderstandings, and treatment benefits [18, 19, 103]. Best practices could be further elucidated through multidisciplinary collaborations in the future.

### Limitations

This research originates from one inpatient treatment center in the Northeast United States and may not be generalizable across cultures and contexts. The center uses the medical model of addiction, they report there is no psycho-educational programming explicitly discussing genetic factors, however informal discussions among residents or between staff and residents may occur prior to our interviews and clients are also likely to have been engaged in recovery elsewhere at different points in their substance use recovery. Service users engaged in substance use treatment at centers employing harm reductionist approaches to behavioral intervention might exhibit more varied responses. Our research is undertaken with individuals with more severe substance use disorders requiring extended inpatient treatment. Thus, this sample may not be representative of individuals with SUD across North America. This sample’s attachment interview was focused on the attachment of mothers and their most recently born child, who was under the age of five at the time of the interview. Therefore, mothers’ concerns about their children’s development may be expressed differently for older children. Furthermore, this unexpected finding came from a secondary analysis not designed to specifically probe the contours of these complex developmental processes thoroughly; therefore, it must be considered cautiously as a call for closer examination in additional samples in both clinical and prospective longitudinal samples.

## Conclusion

Client perceptions of risk factors for intergenerational transmission of addiction are an important consideration in SUD treatment, particularly for parents in treatment. More nuanced understandings of polygenic risk, epigenetics, and biopsychosocial models may be misinterpreted or misunderstood by individuals with SUDs and staff alike. Ultimately people’s understanding of the etiology of addictive behaviors can impact not only clients directly via moderating their feelings of shame or stigma, but also may affect attachment, parenting decisions and familial relationships. Furthermore, given the potential for cultural or contextual insensitivity, any discussions surrounding ‘addiction genes,’ which anecdotally are commonplace in congregate care and treatment settings, have important ethical considerations, and therefore guidelines need to be developed in concert with relevant stakeholders to alleviate unnecessary anxiety, address misconceptions, and ensure best practices are sustained. Finally, the role that attachment style plays in these individuals’ conceptions and anxieties about the transmission of substance use appears important in these data, and these issues should be further explored in future research.

## Data Availability

The datasets generated and/or analysed during the current study are not publicly available due to client confidentiality and agreements. Reasonable requests to access data may be made to Dr. Emily Bosk.
